# Rapid Plasma Electrolytic Oxidation Synthesis of Intermetallic PtBi/MgO/Mg Monolithic Catalyst for Efficient Removal of Organic Pollutants

**DOI:** 10.3390/ma17030605

**Published:** 2024-01-26

**Authors:** Jiayi Rong, Mengyang Li, Feng Cao, Qianwei Wang, Mingran Wang, Yang Cao, Jun Zhou, Gaowu Qin

**Affiliations:** Key Lab for Anisotropy and Texture of Materials (MoE), School of Materials Science and Engineering, Northeastern University, Shenyang 110819, China; 2210214@stu.neu.edu.cn (J.R.); 2310170@stu.neu.edu.cn (M.L.); 2270554@stu.neu.edu.cn (Q.W.); 2200639@stu.neu.edu.cn (M.W.); 2270359@stu.neu.edu.cn (Y.C.); hafouniu@126.com (J.Z.)

**Keywords:** plasma electrolytic oxidation, PtBi, intermetallic compounds, monolithic catalyst, removal of organic pollutants

## Abstract

The intermetallic PtBi/MgO/Mg monolithic catalyst was first prepared using non-equilibrium plasma electrolytic oxidation (PEO) technology. Spherical aberration-corrected transmission electron microscope (ACTEM) observation confirms the successful synthesis of the PtBi intermetallic structure. The efficiency of PtBi/Mg/MgO catalysts in catalyzing the reduction of 4-nitrophenol (4-NP) to 4-aminophenol (4-AP) in the presence of NaBH_4_ was demonstrated. The activity factor for the catalyst is 31.8 s^−1^ g^−1^, which is much higher than reported values. In addition, the resultant catalyst also exhibits excellent catalytic activity in the organic pollutant reaction of p-nitrobenzoic acid (p-NBA) and methyl orange (MO). Moreover, benefiting from ordered atomic structures and the half-embedded PtBi nanoparticles (NPs), the catalyst demonstrates excellent stability and reproducibility in the degradation of 4-NP. This study provides an example of a simple method for the preparation of intermetallic structures as catalysts for organic pollutant degradation.

## 1. Introduction

Over the past few decades, nitroaromatic compounds and organic dyes, due to their inherent toxicity, mutagenicity, teratogenicity, and carcinogenic potential, have drawn considerable attention internationally [1,2,3]. Catalytic reduction or conversion of pollutants into practical chemical products is economical. For example, the conversion of 4-NP into 4-AP is a key intermediate in pharmaceutical production [4]. Noble metal NPs, such as Ag, Pt, and Au, have been proved as efficient catalysts with high activity in the catalytic reduction of nitroarene [5,6,7]. At present, the most widely used for catalytic hydrogenation are platinum (Pt)-based materials, but the widespread application of Pt is constrained by high prices. Addressing the dilemma of restrained resources of Pt, Pt-based bimetallic catalysts have constituted a plausible alternative [8,9,10]. Pt-based nanoalloy powder catalysts have significant catalytic activity, but they often face the problems of agglomeration, inactivation, and difficult recovery in liquid phase catalysis [11]. Moreover, these disordered solid solution catalysts encounter challenges regarding restricted reaction kinetics and the inevitable leaching of non-noble metals. Compared with the traditional powder catalysts, the monolithic catalyst has the advantages of well-developed pore structure, uniform dispersion of active components on the support, and high recyclability [12,13].

Our team has successfully developed an innovative approach using PEO technology to construct Au/MgO/Mg monolithic catalysts. These catalysts have demonstrated excellent catalytic activity and thermal stability in reaction to the hydrogenation of 4-nitrophenol reactions [14]. Monolithic catalysts with other noble metals and their alloys as active sites were also subsequently prepared [15]. However, the current focus is primarily on disordered alloys, and further research is urgently needed in the field of ordered intermetallic compounds.

Bismuth (Bi) with high electronegativity, esteemed as an exceptional promoter of p-electron metal, has garnered considerable attention in the field of PtBi intermetallic compound design. Xie advanced a facile wet-chemical synthesis of heterogeneous fcc-Pt/hcp-PtBi thick-edge nanoplates (PtBi NPs) [16]. Owing to their unique structure and heterogeneous feature, these PtBi NPs exhibited enhanced catalytic activity and stability for the oxidation of formic acid in comparison to commercial Pt/C. Dou synthesized a monatomic Pt layer (Pt-skin) on ordered intermetallic PtBi clusters (PtBi@Pt) supported on graphene via a single atom self-assembling (SAS) method to form a superior catalyst [17]. It can achieve a highly efficient and complete electrochemical oxidation pathway to CO_2_/CO_3_^2−^. The enhancement effect of Bi on catalytic performance was attributed to the following three points: (1) Site isolation effect. The formation of geometric blocking on noble metals by Bi that diminished the size of active site ensembles and regulated intermediates’ adsorption energy of Pt. In ordered intermetallic compounds, the Pt–Pt spacing can be adapted up to twice that measure. For instance, on the (001) plane of PtBi, the Pt–Pt distance constitutes 4.32 Å [18]. (2) Enhanced electronic effects. High affinity of Bi for oxygen prevented the noble metal from over-oxidation and maintained its metallic state [19]. (3) Excellent stability. An elevated formation energy in hcp-PtBi contributes to both enhanced interatomic interactions and a heightened resistance to etching processes [20,21]. Therefore, the development of a PtBi intermetallic monolithic catalyst with efficient organic pollutant removal can be of great significance in reducing environmental pollution.

Herein, we report a simple and effective method for preparing half-embedded PtBi intermetallic monolithic catalysts using PEO. The atomically resolved scanning transmission electron microscopy (STEM) indicates that the intermetallic PtBi NPs of about 6.6 nm in size are successfully synthesized. The as-synthesized intermetallic PtBi/MgO/Mg displays outstanding organic pollutant-removal performances for 4-NP, p-NBA, and MO. The activation energy (E_a_) and activity factor (K) of the catalytic 4-NP reduction reaction were calculated to be E_a_ = 17.99 kJ mol^−1^ and K = 31.8 s^−1^ g^−1^, outperforming those of most reported noble metal-based catalysts. In addition, the catalysts can achieve complete conversion of p-NBA and MO within 6 min and 60 min, respectively. The resulting intermetallic PtBi/MgO/Mg monolithic catalyst exhibited exceptional cycle stability and thermal stability due to the fact that particles are half-embedded. Even after 50 cycles, the conversion rate remains above 90%, and the catalyst can be successfully recovered after regeneration for long-term durability. After heat treatment at 900 °C, there was no agglomeration and no phase transformation of intermetallic PtBi NPs. PtBi/MgO/Mg monolithic catalysts exhibited excellent stability and catalytic activity, providing a new perspective for exploring the preparation process of monolithic catalysts and expanding their applications in the field of heterogeneous catalysis in the future.

## 2. Materials and Methods

### 2.1. Materials

Magnesium bulks (99.99%) were purchased from Suzhou Xinghai Electronic Commerce Co., Ltd., Suzhou, China. Chloroplatinic acid (H_2_PtCl_6_·6H_2_O) was purchased from Zhongtian Dine Chemical Co., Ltd., Zhoushan, China. Bismuth acetate (Bi (C_2_H_3_O_2_)), sodium borohydride (NaBH4), and p-nitrophenol (4-NP) were obtained from Aladdin, Shanghai, China. Methyl orange (MO) was purchased in the Tianjin Tianxin Fine Chemical Development Center, Tianjin, China. P-nitrobenzoic acid (p-NBA) and ethylenediaminetetraacetic acid disodium salt (EDTA-2Na) were purchased from Tianjin Damao Chemical Reagent Factory, Tianjin, China. Potassium hydroxide (KOH), sodium metasilicate nonahydrate (Na_2_SiO_3_·9H_2_O), and potassium fluoride (KF) were purchased from Sinopharm Chemical Reagent Co., Ltd, Shanghai, China. All the reagents are analytical reagents (ARs).

### 2.2. Characterization

X-ray diffraction (XRD) (Smart Lab, Rigaku, Osaka city, Japan) was used to observe the phase structure and chemical composition of the coating by scanning the sample with Cu Kα radiation at a scanning rate of 4°/min in the 2θ range of 20° and 90°. A field emission scanning electron microscope (FESEM) (JSM-7001F, Tokyo, Japan) was used to observe and characterize the surface and cross-sectional morphology of the coating. The semi-quantitative chemical composition of the coatings was examined using energy dispersive spectroscopy (EDS) incorporated into the SEM. The morphology, distribution, and dispersion of PtBi nanoparticles were characterized using transmission electron microscope (TEM) (JEM-2100F, Tokyo, Japan). The preparation process of TEM samples is as follows: A small amount of powder is taken from the monolithic catalyst surface coating and dispersed in ethanol. Then, 5 μL of solution is dispersed on the carbon-supporting coated copper mesh, dried, and characterized. An inductively Coupled Plasma-Optical Emission Spectrometer (ICP-OES) was used for the composition detection of Pt (Waltham, MA, USA).

### 2.3. Pretreatment of Mg Plates

The commercially available magnesium block was processed into a 40 mm × 20 mm × 2 mm magnesium plate (with the surface area of 18.4 cm^2^) via wire cutting, and an electric drill was used to drill a hole in a corner of the magnesium plate with an aperture of about 2 mm. A metallographic polishing machine was used to grind the Mg plates. Then, 600 # sandpaper was used with clean water to coarsely grind the sample on a metallographic grinding and polishing machine to remove surface stains. The samples were finely ground with 2000 # sandpaper ethanol to remove fine scratches on the surface. The ethanol was continuously added during the process to prevent the sample from being oxidized. Finally, the Mg plates were rinsed with alcohol and stored in alcohol.

### 2.4. Preparation of the PtBi/MgO/Mg Catalyst

PtBi/MgO/Mg catalysts were fabricated using a rapid PEO process. Prior to the experiment, a pretreated magnesium plate and a stainless-steel plate were selected as the anode and cathode, respectively. Oxide coatings were prepared via immersion of the whole Mg plate in an electrolyte solution (8 g L^−1^ Na_2_SiO_3_·9H_2_O, 7 g L^−1^ KOH, 5 g L^−1^ KF, 0.1 mmol L^−1^ Bi(C_2_H_3_O_2_)_3_, 0.1 mmol L^−1^ H_2_PtCl_6_·6H_2_O, and 0.4 mmol L^−1^ EDTA-2Na in 500 mL water), and processed under 400 V DC-applied voltage for 30 s. MgO support and PtBi nanoparticles were created simultaneously due to local high temperature and pressure derived from the discharge. Finally, the samples were rinsed three times with deionized water and dried in a vacuum at 25 °C for 1 h. 

### 2.5. Organic Pollutant Catalytic Removal Experiments

In this work, 10 mL of 0.1 M NaBH_4_ solution was used as the hydrogen source for the catalytic reactions, and 10 mL of 0.1 mM reactant (4-NP, p-NBA, or MO) was used. The monolithic catalyst composed of Mg matrix, MgO layer, and metal active substance was completely immersed in the solution by stirring at a fixed temperature in a water bath (the rotating speed was set to 40 r/s), and 2.5 mL aqueous solution was taken each time for ultraviolet–visible spectrophotometer to measure its spectrum. When the test was conducted using the UV-spectrophotometer, we lifted the monolithic catalyst to stop the reaction and stopped the rotation of the water bath. After the test process, the 2.5 mL solution and the monolithic catalyst were both put back and timed for the next period. All kinds of substances have different molecules and groups, so their absorption of light energy will not be the same, corresponding to different light absorption lines. According to the Lambert–Beer law, the absorbance is proportional to the concentration of solution in dilute solution.

T = I/I_0_ = 10^−*k*bC^; A = −lg T = −lg (I/I_0_) = lg (1/T) = *k*bC

For the activation energy of the catalytic reaction, the temperature of the reaction solution was adjusted to 25 °C, 35 °C, and 45 °C, respectively, and the reaction rate constants (*k*) were measured, and the activation energy (E_a_) was calculated via fitting. The relationship between k and temperature could be expressed using the Arrhenius equation as follows:

ln *k* = ln A − E_a_/RT

where A represents the Arrhenius factor, R is the universal gas constant (R = 8.314), and T is the corresponding reaction temperature (T = 25 °C, 35 °C, or 45 °C).

To evaluate the activity of the PtBi/MgO/Mg monolithic catalyst, activity factor *K* is induced. Before that, the mass of active sites must be calculated as follows:

m_active sites_ = m_Pt content_ × area

In the above formula, the ‘area’ refers to the surface area of the Mg plate, which is calculated to be 18.4 cm^2^ (4 cm × 2 cm × 2 + 4 cm × 0.2 cm × 2+ 0.2 cm × 2 cm × 2 = 18.4 cm^2^).

To evaluate the durability of the PtBi/MgO/Mg monolithic catalyst, the following steps can be adopted: After the reaction, the catalyst is taken out, rinsed three times with deionized water, and dried in a vacuum to enter the next catalytic cycle reaction. 

To evaluate the reproducibility of the PtBi/MgO/Mg monolithic catalyst, the following steps can be adopted: After multiple cycles, the catalyst is taken out and washed. The heat treatment process is repeated. The PtBi/MgO/Mg monolithic catalyst is reduced to 5% H_2_/Ar atmosphere at 400 °C for 2 h.

## 3. Results and Discussion

**Figure 1:**
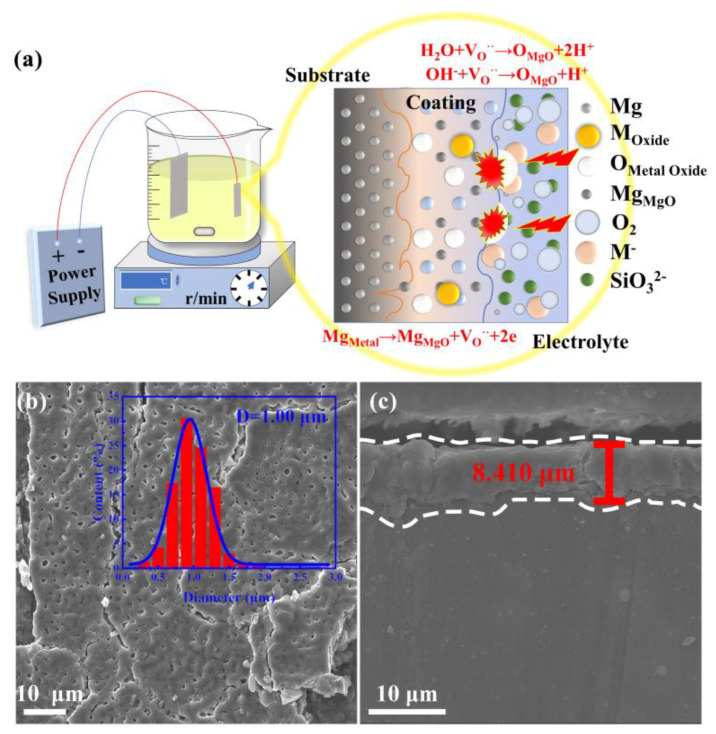
Fabrication and morphology of intermetallic PtBi/MgO/Mg: (**a**) Schematic illustration of the fabrication of intermetallic PtBi/MgO/Mg; (**b**) Surface morphology of intermetallic PtBi/MgO/Mg in which the inset is the pore-size distribution diagram; (**c**) SEM cross−sectional image of the intermetallic PtBi/MgO/Mg.

The intermetallic PtBi/MgO/Mg were synthesized via a facile PEO method with subsequent heat treatment. Bimetallic PtBi NPs with varying ratios were also prepared by simply adjusting the ratio of Pt/Bi precursors before PEO progress. The schematic representation of the substrate/coating/electrolyte system in the PEO process is shown in Figure 1a. At the interface of the substrate and coating, Mg^2+^ reacts with the anions, forming compounds and creating new anion vacancies in the coating [22]. At the interface of the electrolyte and coating, metal-containing anion precursors in the electrolyte are implanted into the coating, occupying anion vacancies under the influence of an electric field [23]. In this non-equilibrium process involving local high temperature and pressure, NPs with uniform composition are easily formed in situ within MgO supports, despite varying reduction rates of Pt^4+^ and Bi^3+^. The subsequent heat treatment can reduce the oxidized metal species into the intermetallic NPs. To gain a deeper understanding of the composition-dependent catalytic mechanism, bimetallic Pt/Bi NPs with varying ratios were also prepared by simply adjusting the ratio of Pt/Bi precursors.

The X-ray diffraction (XRD) patterns of the resultant sample were indexed to Mg (PDF # 35-0821) and MgO (PDF # 45-0946). The XRD does not show the typical diffraction peaks of intermetallic PtBi owing to the low concentration (Figure S1). The scanning electron microscopy (SEM) image of the intermetallic PtBi/MgO/Mg exhibits uniform volcanic pores of approximately 1 μm at the surface (Figure 1b). This porous architecture ensures a high specific surface area for the MgO layer as a catalyst support. The coating of the MgO layer shows a thickness of about 8 μm and exhibits a tight metallurgical bond with the Mg matrix, owing to the unique high-voltage plasma effect. This can effectively prevent the coating from peeling off the surface of the substrate (Figure 1c and Figure S2). The surface mainly contains MgO and traces Si and F (Table S1). The presence of the Si and F elements is attributed to the decomposition of electrolyte species via discharges. The exact noble metal content was determined to be 2.8 μg cm^−2^ using ICP-OES.

**Figure 2:**
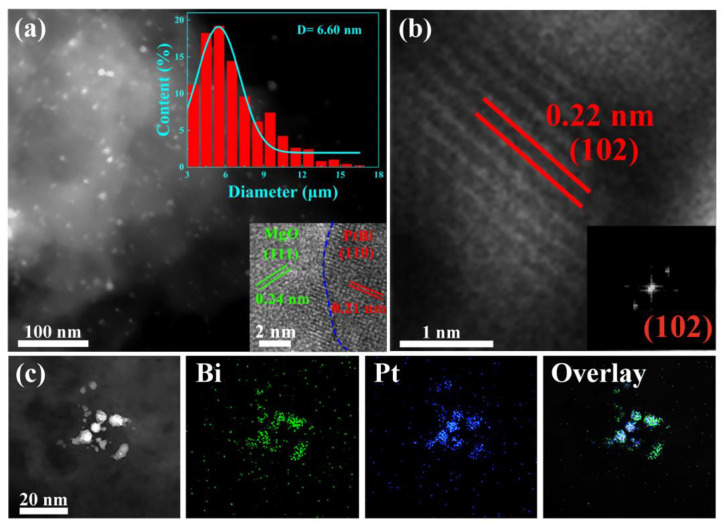
(**a**) HAADF-STEM image of the intermetallic PtBi/MgO/Mg catalyst, and insets are the nanoparticle (NP) size distribution diagram and HRTEM of intermetallic PtBi NP; (**b**) HRTEM image of intermetallic PtBi NPs, and inset is the corresponding FFT pattern; (**c**) The STEM image of intermetallic PtBi NPs and STEM-mapping images of Bi and Pt and overlay of Bi and Pt.

The high-angle annular dark-field scanning transmission electron microscopy (HAADF-STEM) image shows that the ultrasmall intermetallic PtBi NPs (6.6 nm) are homogeneously located on the MgO (Figure 2a). During PEO process, due to its unique high temperature and high-pressure plasma effect, catalyst precursors H_2_PtCl_6_ and Bi (C_2_H_3_O_2_)_3_ were decomposed and reduced to PtBi nanoparticles, which were loaded in situ into the porous MgO layer that formed simultaneously [24]. And they were reduced to alloy nanoparticles in subsequent heat treatment. The magnified image shows that the PtBi NPs are partially encapsulated into the support (inset of Figure 2a). Such a structure can inhibit PtBi particle migration and coalescence. ACTEM was performed to further analyze the structure of the nanoparticles. The high-resolution STEM image and the corresponding FFT image reveal a lattice spacing of 0.22 nm in Figure 2b and its inset, corresponding to the (102) plane of intermetallic PtBi (PtBi: ICSD # 58845) [17]. Further energy-dispersive X-ray (EDX) elemental mapping analysis demonstrates the simultaneous presence and homogeneous dispersion of Pt and Bi, indicating the uniform composition of the ultrasmall nanoparticles (Figure 2c). For comparison, Pt/MgO/Mg was also synthesized in a similar procedure. The 0.23 nm lattice spacing is consistent with the (111) lattice spacing of the face-centered cubic (fcc) crystal structure of Pt (PDF#87-0640) (Figure S3). 

**Figure 3:**
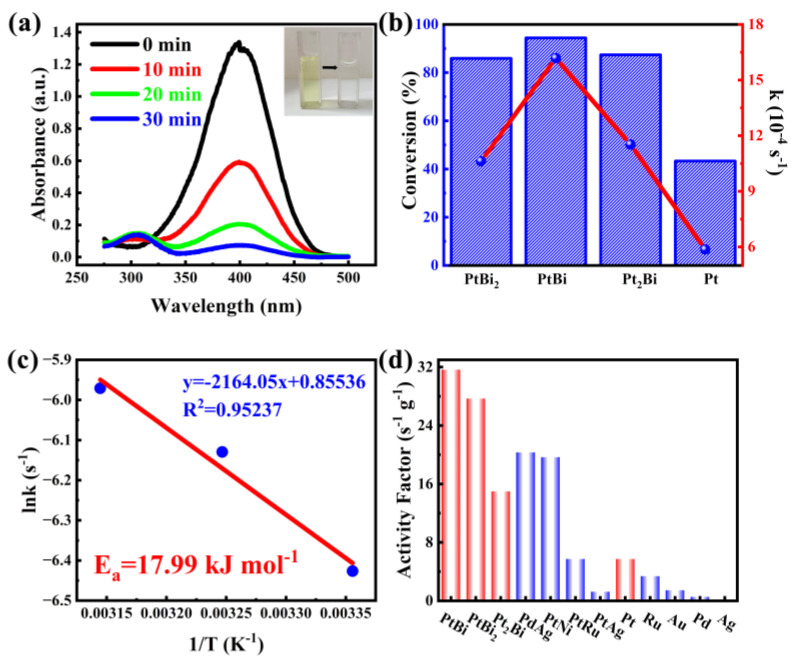
(**a**) Time−dependent UV−visible absorption spectra of 4-NP catalytic removal by intermetallic PtBi/MgO/Mg catalyst, and the inset is the corresponding color change picture; (**b**) Conversion and equilibrium constant k of 4-NP catalytic removal by catalysts with diverse metal ratios of Pt and Bi in 30 min; (**c**) The Arrhenius plot of ln *k* vs. 1000/T for the catalytic reaction; (**d**) The activity factors of intermetallic PtBi/MgO/Mg catalyst and other reported noble metal catalysts.

To evaluate the catalytic performance of the as-prepared intermetallic PtBi/MgO/Mg for organic pollutant degradation reaction, 4-nitrophenol (4-NP) is first chosen as a probe molecule. In the absence of a catalyst, the shape and peak intensity of the absorption spectra remained essentially unchanged after 12 h, indicating that the reduction of 4-NP was kinetically limited (Figure S4). In the presence of intermetallic PtBi/MgO/Mg, the unique absorption peak of 4-NP at 400 nm gradually decreased, and a new band associated with 4-aminophenol (4-AP) appeared at 296 nm (Figure 3a). The intensity of the color gradually decreased with time from yellow to colorless. When the intermetallic PtBi/MgO/Mg was added, 4-NP was nearly completely (96.9%) converted in 30 min, significantly better than Pt/MgO/Mg (43.3%), disordered alloy Pt_2_Bi/MgO/Mg (87.4%), and PtBi_2_/MgO/Mg (85.9%) (Figure 3b and Figure S5). The E_a_ value of the 4-NP catalytic reduction reaction of hcp-PtBi/MgO/Mg was calculated to be 17.99 kJ mol^−1^, which showed good agreement with the Arrhenius equation (R^2^ = 0.95237) in Figure 3c. The E_a_ of hcp-PtBi/MgO/Mg is smaller than the E_a_ observed for other noble metal-based catalysts reported in the literature (Table S2) [25,26]. To further illustrate the utilization rate of metal atoms, the activity factor, denoted as K = *k*/m_active site_, was calculated. The K of PtBi/MgO/Mg was calculated as 31.8 s^−1^ g^−1^, which is much higher than those reported surpassing a majority of reported noble metal catalysts (Figure 3d) [27,28]. In addition, the activity factors of stoichiometric ratios PtBi_2_ (28 s^−1^ g^−1^) and PtBi (31.8 s^−1^ g^−1^) are also much higher than those of disordered Pt_2_Bi (15 s^−1^ g^−1^) and single metal Pt (6 s^−1^ g^−1^). These results clearly suggest that PtBi/MgO/Mg has an ultra-high utilization of Pt atoms thanks to the porous intermetallic structure and small particle size of the monolithic catalyst coating.

To investigate whether the superior catalytic performance of the hcp-PtBi/MgO/Mg catalyst is general in the nitroarenes reduction, we have also explored the removal of several other organic pollutants, including p-nitrobenzoic acid (p-NBA) and methyl orange (MO). We found that hcp-PtBi/MgO/Mg exhibited efficient catalytic performance toward all corresponding organic pollutants. For p-NBA reduction reaction, the intermetallic PtBi/MgO/Mg catalyst exhibited the shortest degradation time of 6 min compared to the others with varying ratios (19 min for PtBi_2_, 19 min for Pt_2_Bi, and 18 min for Pt), as shown in Figure S6. For MO reduction reaction, MO was nearly completely converted in 60 min in the presence of intermetallic PtBi/MgO/Mg (Figure S7), significantly better than Pt/MgO/Mg, disordered alloy Pt_2_Bi/MgO/Mg, and PtBi_2_/MgO/Mg.

**Figure 4:**
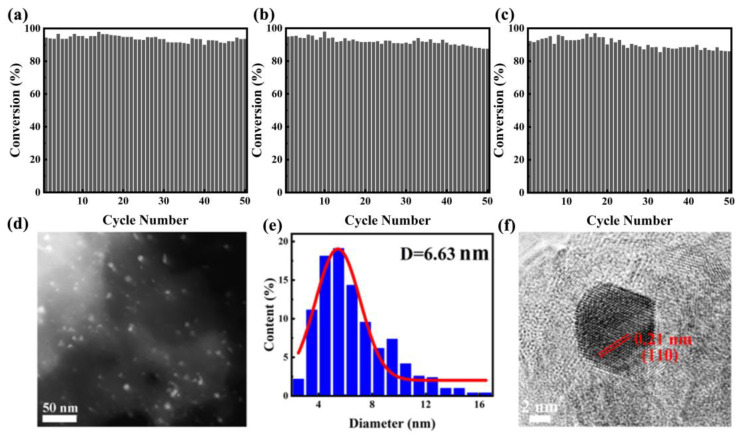
(**a**) Evaluation of the durability of intermetallic PtBi/MgO/Mg catalyst for the 4-NP catalytic removal. The reproducibility of the (**b**) first regeneration after 70 cycles; (**c**) second regeneration after another 60 cycles; (**d**) HAADF-STEM image after cycles; (**e**) NPs size distribution diagram; and (**f**) HRTEM image after cycles.

The stability and reproducibility of catalysts play crucial roles in determining their performance as heterogeneous catalysts, particularly in industrial and large-scale applications. It is convenient to separate the monolithic catalyst from the reaction mixture after a catalytic run. We also tested the recycling stability of PtBi/MgO/Mg with 4-NP reduction as the model reaction (Figure 4a). After the reaction, the structure catalyst recovered with simple deionized water washing. The recovered catalyst was directly used for the next run. PtBi/MgO/Mg can be stably recycled for 50 cycles without obvious loss of catalytic activity. Intermetallic PtBi/MgO/Mg prepared using PEO demonstrated superior performance compared to previously reported catalysts using other methods (Table S3) [29,30,31,32]. Furthermore, to confirm the reversibility of the catalyst, a heat treatment reduction process was performed after repeated cycles. Specifically, the catalyst was calcined in a 5% H_2_/Ar atmosphere for 2 h. After the first regeneration, the catalyst was converted to 87.5% after 50 cycles (Figure 4b). After the second regeneration, the conversion rate of the 50th reaction remained at 86% (Figure 4c), indicating that the catalyst has amazing regenerative capacity. Additionally, thanks to the half-embedded structure, the average size of the PtBi NPs did not increase, and no agglomeration was observed (Figure 4d,e). HRTEM characterization revealed a crystal plane spacing of 0.21 nm corresponding to the (110) of the PtBi, which indicated the intrinsic catalytic stability of IMs (Figure 4f). Therefore, the cyclic test provided strong evidence supporting the excellent cycle stability and reproducibility of the monolithic catalyst intermetallic PtBi/MgO/Mg.

To confirm the thermal stability and sintering resistance of the catalyst, we subjected the as-prepared intermetallic PtBi/MgO/Mg monolithic catalyst to high-temperature calcination. After calcination at 600 °C in Ar for 2 h, as shown in Figure S8, the value of k (11.97 × 10^−4^ s^−1^) did not exhibit a significant decrease. Considering the low melting point of the Mg substrate, we removed the surface oxide coating and further heat-treated the catalyst at 900 °C for 2 h. HAADF-STEM characterization results (Figure S9) revealed that the size and monodispersity of PtBi NPs on the porous MgO support remained remarkably unchanged. The sintering and coarsening of PtBi NPs on the MgO support surface were significantly inhibited by the unique embedded structure and the presence of oxygen vacancies. The embedded structure ensures the immobilization of NPs on the surface of the carrier and limits agglomeration. The oxygen vacancies consistently maintained the metallic state of the NPs and prevented oxidation. Thus, all the evidence confirms the superior thermal stability of the intermetallic PtBi/MgO/Mg catalyst.

## 4. Conclusions

In summary, the monodispersed PtBi intermetallic monolithic catalyst was successfully synthesized through a rapid PEO process. HAADF-STEM shows that the NPs are partially embedded in MgO, which generates a strong interaction between NPs and MgO. ACTEM characterization confirms the formation of the ordered PtBi NPs. The intermetallic PtBi/MgO/Mg monolithic catalyst showed universal and excellent catalytic activity in the reduction of organic pollutants (4-NP, p-NBA, and MO). Due to the embedding and intermetallic structure, the catalyst exhibits excellent cycling and thermal stability. This paper innovatively proposes the rapid preparation of ordered intermetallic compounds using the PEO method. It broadens the idea for the preparation of ordered intermetallic compounds and fills the gap of PtBi via the PEO method. The PtBi/MgO/Mg monolithic catalyst has good stability and catalytic activity, which sets a solid foundation for exploring this new monolithic catalyst synthesis process and expanding its application in the field of multi-phase catalysis.

## Data Availability

All data generated for this work are included in this article.

## Supplementary Materials

The following supporting information can be downloaded at: https://www.mdpi.com/article/10.3390/ma17030605/s1, Figure S1: XRD patterns of (a) PtBi/MgO/Mg monolithic catalyst, (b) PtBi_2_/MgO/Mg, Pt_2_Bi/MgO/Mg and Pt/MgO/Mg monolithic catalysts. Figure S2: Low-magnification SEM cross-sectional image of the intermetallic PtBi/MgO/Mg. Figure S3: HRTEM image of Pt/MgO/Mg NPs, and the inset is the corresponding FFT pattern. Figure S4: Time-dependent UV−visible absorption spectra of 4-NP catalytic removal without any catalyst. Figure S5: Time-dependent UV−visible absorption spectra of 4-NP catalytic removal via PtBi/MgO/Mg monolithic catalyst with diverse metal ratios: (a) PtBi_2_; (b) Pt_2_Bi; (c) Pt.; (d) Rate curve and linear fitting kinetic curve of 4-NP catalytic removal (e) via catalysts with diverse metal ratios of Pt and Bi; (f) by the hcp-PtBi/MgO/Mg monolithic catalyst at diverse temperatures. Figure S6: Time-dependent UV−visible absorption spectra of p-NBA catalytic removal via PtBi/MgO/Mg monolithic catalyst with diverse metal ratios: (a) Bi; (b) PtBi_2_; (c) PtBi; (d) Pt_2_Bi; (e) Pt.; (f) Degradation time of MO catalytic removal via catalysts with diverse metal ratios. Figure S7: Time-dependent UV−visible absorption spectra of MO catalytic removal via monolithic catalyst with diverse metal ratios: (a) Bi; (b) PtBi_2_; (c) PtBi; (d)Pt_2_Bi; (e) Pt.; (f) Rate curve and (g) linear fitting kinetic curve of MO catalytic removal via catalysts with diverse metal ratios of Pt and Bi. (h) Conversion and equilibrium constant *k* of MO catalytic removal via catalysts with diverse metal ratios in 60 min. Figure S8: (a) Time-dependent UV−visible absorption spectra of 4-NP catalytic removal via PtBi/MgO/Mg monolithic catalyst treated with calcination at 600 °C for 2 h in Ar. (b) The corresponding rate curve and (c) linear fitting kinetic curve. Figure S9: HAADF-STEM images of PtBi/MgO/Mg monolithic catalyst treated with (a) direct calcination at 600 °C and (b) powder calcination at 900 °C for 2 h in Ar. (c) and (d) their corresponding NP size distribution diagrams. Table S1: The EDS results of PtBi/MgO/Mg monolithic catalyst. Table S2: The apparent activation energy (E_a_) for 4-NP catalytic removal using other catalysts containing noble metals reported in other works. Table S3: The recyclability of the PtBi/MgO/Mg catalyst and other catalysts prepared using various methods in other works.
