# Supplementary material for: Rapid Plasma Electrolytic Oxidation Synthesis of Intermetallic PtBi/MgO/Mg Monolithic Catalyst for Efficient Removal of Organic Pollutants
Source: Materials (Basel). 2024 Jan 26;17(3):605. doi: 10.3390/ma17030605 (PMC10856466; doi:10.3390/ma17030605)
Supplement: Supplementary file 1 [file materials-17-00605-s001.zip › materials-2820459-supplementary-done.pdf]

materials

MDPI

Article

Rapid Plasma Electrolytic Oxidation Synthesis of Intermetallic PtBi/MgO/Mg Monolithic Catalyst for Efficient Removal of Organic Pollutants

Jiayi Rong †, Mengyang Li †, Feng Cao *, Qianwei Wang, Mingran Wang, Yang Cao, Jun Zhou and Gaowu Qin *

Key Lab for Anisotropy and Texture of Materials (MoE), School of Materials Science and Engineering, Northeastern University, Shenyang 110819, China; 2210214@stu.neu.edu.cn (J.R.); 2310170@stu.neu.edu.cn (M.L.); 2270554@stu.neu.edu.cn (Q.W.); 2200639@stu.neu.edu.cn (M.W.); 2270359@stu.neu.edu.cn (Y.C.); hafouniu@126.com (J.Z.)

* Correspondence: caof@atm.neu.edu.cn (F.C.); qingw@smm.neu.edu.cn (G.Q.)

† These authors contributed equally to this work.

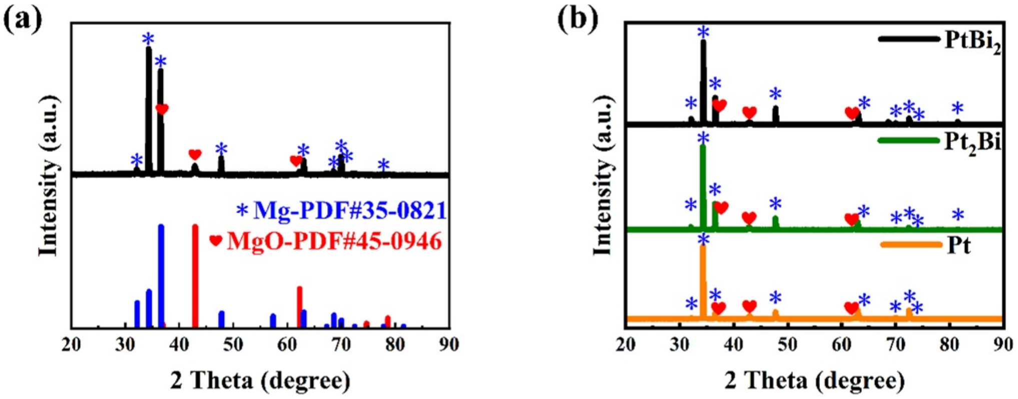

Figure S1. XRD patterns of (**a**) hcp-PtBi/MgO/Mg monolithic catalyst, (**b**) $PtBi_2$/MgO/Mg, Pt_2Bi/MgO/Mg and Pt/MgO/Mg mono-lithic catalysts. The blue ❇ symbol presents the characteristic peak of Mg-PDF#35-0821. The red ♥ symbol presents the characteristic peak of MgO-PDF#45-0946.

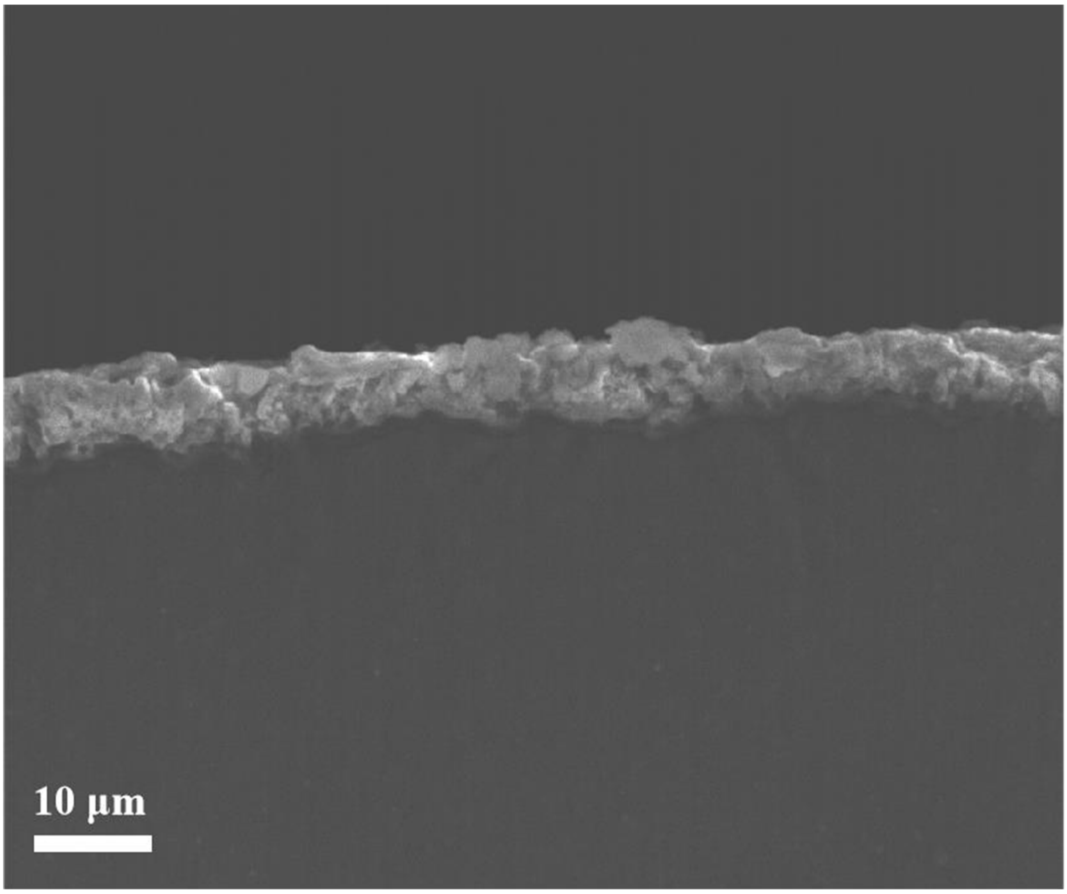

Figure S2. Low-magnification SEM cross-sectional image of the intermetallic PtBi/MgO/Mg.

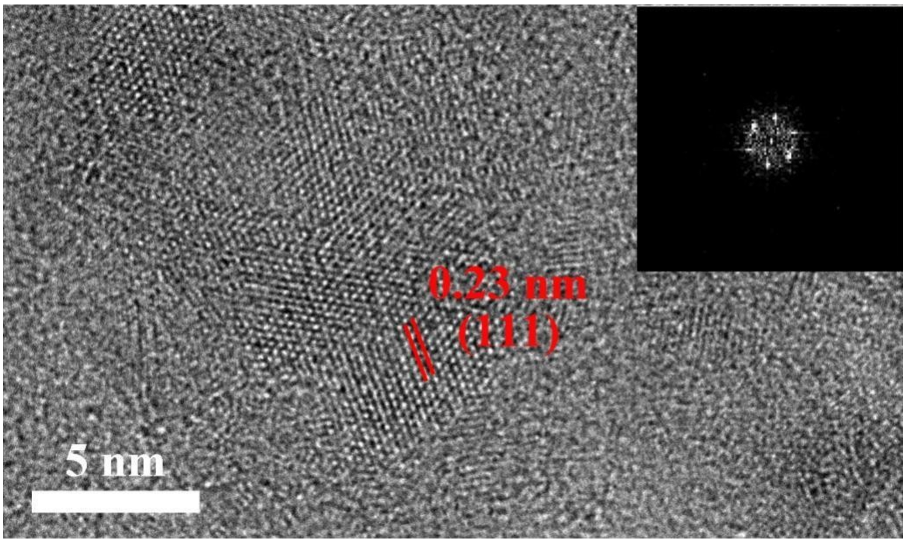

Figure S3. HRTEM image of fcc-Pt/MgO/Mg NPs; and the inset is the corresponding FFT pattern.

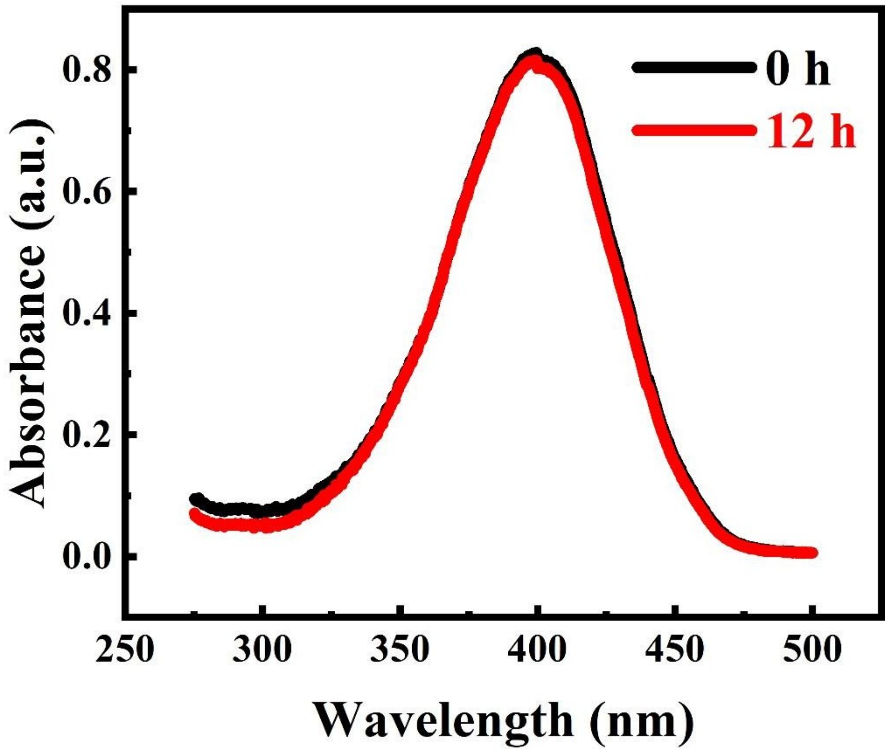

Figure S4. Time-dependent UV-visible absorption spectra of 4-NP catalytic removal without any catalyst.

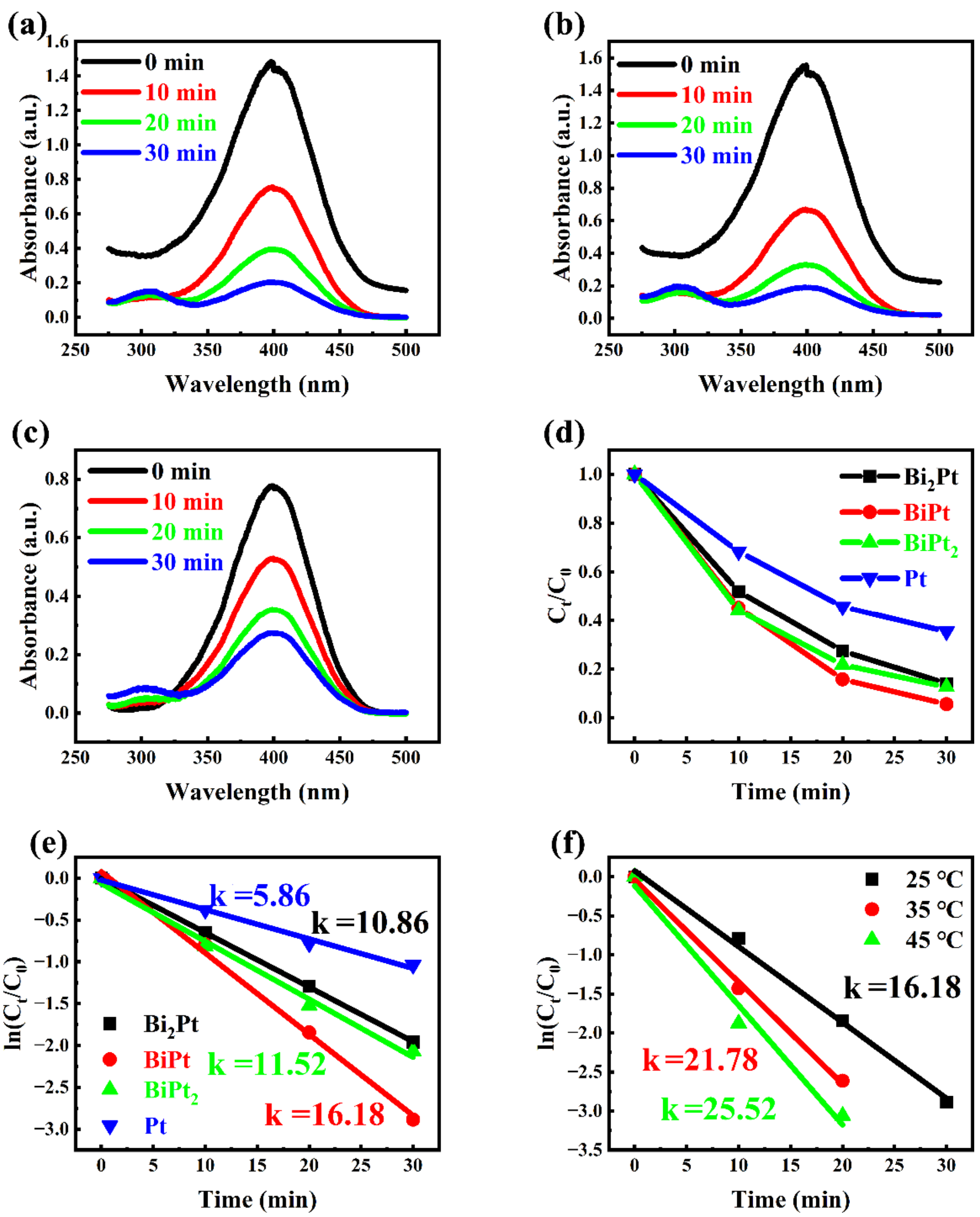

Figure S5. Time-dependent UV-visible absorption spectra of 4-NP catalytic removal by PtBi/MgO/Mg monolithic catalyst with di-verse metal ratios:(**a**) $PtBi_2$; (**b**) Pt_2Bi; (**c**) Pt. (**d**) Rate curve and linear fitting kinetic curve of 4-NP catalytic removal (**e**) by catalysts with diverse metal ratios of Pt and Bi; (**f**) by hcp-PtBi/MgO/Mg monolithic catalyst at diverse temperatures.

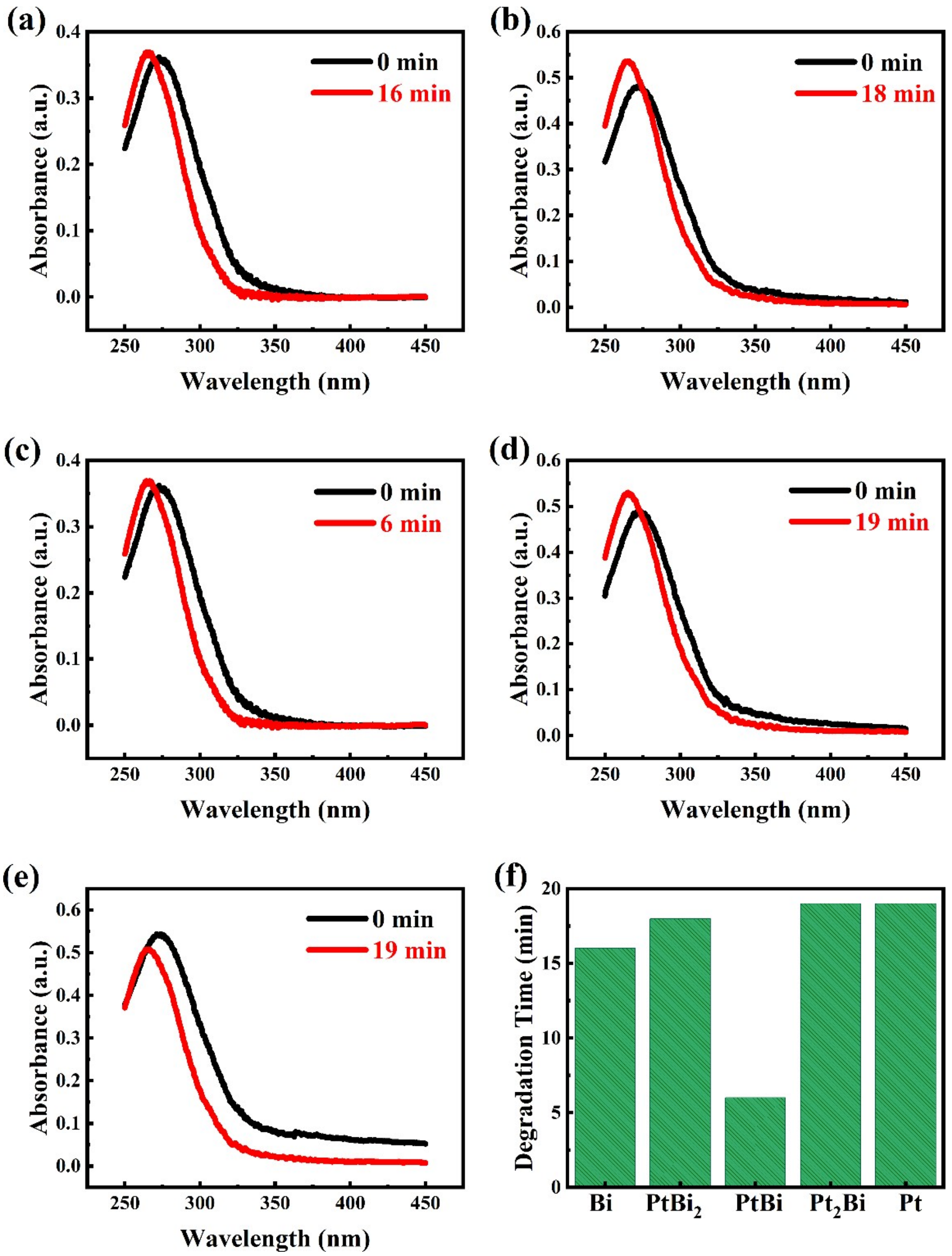

Figure S6. Time-dependent UV-visible absorption spectra of p-NBA catalytic removal by PtBi/MgO/Mg monolithic catalyst with diverse metal ratios:(**a**) Bi; (**b**) $PtBi_2$; (**c**) PtBi; (**d**) Pt_2Bi; (**e**) Pt. (**f**) Degradation time of MO catalytic removal by catalysts with diverse metal ratios.

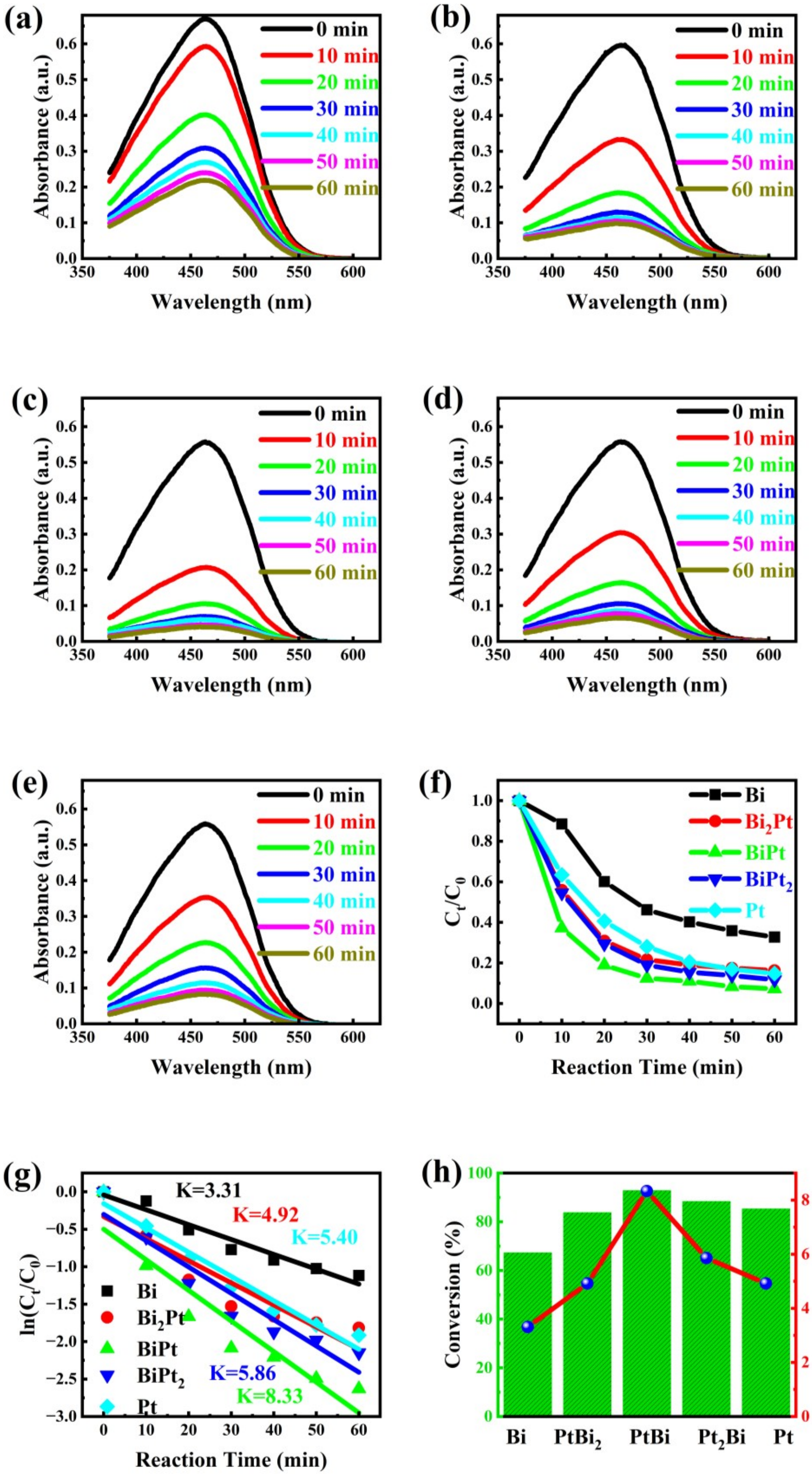

Figure S7. Time-dependent UV-visible absorption spectra of MO catalytic removal by monolithic catalyst with diverse metal ratios: (**a**) Bi; (**b**) $PtBi_2$; (**c**) PtBi; (**d**)Pt_2Bi; (**e**) Pt. (**f**) Rate curve and (**g**) linear fitting kinetic curve of MO catalytic removal by catalysts with diverse metal ratios of Pt and Bi. (**h**) Conversion and equilibrium constant *k* of MO catalytic removal by catalysts with diverse metal ratios in 60 min.

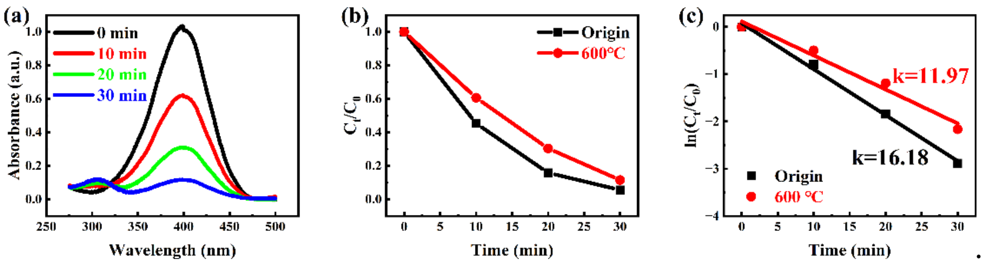

Figure S8. (**a**) Time-dependent UV-visible absorption spectra of 4-NP catalytic removal by hcp-PtBi/MgO/Mg monolithic catalyst treated by calcination at 600 ºC for 2 h in Ar. (**b**) The corresponding rate curve and (**c**) linear fitting kinetic curve.

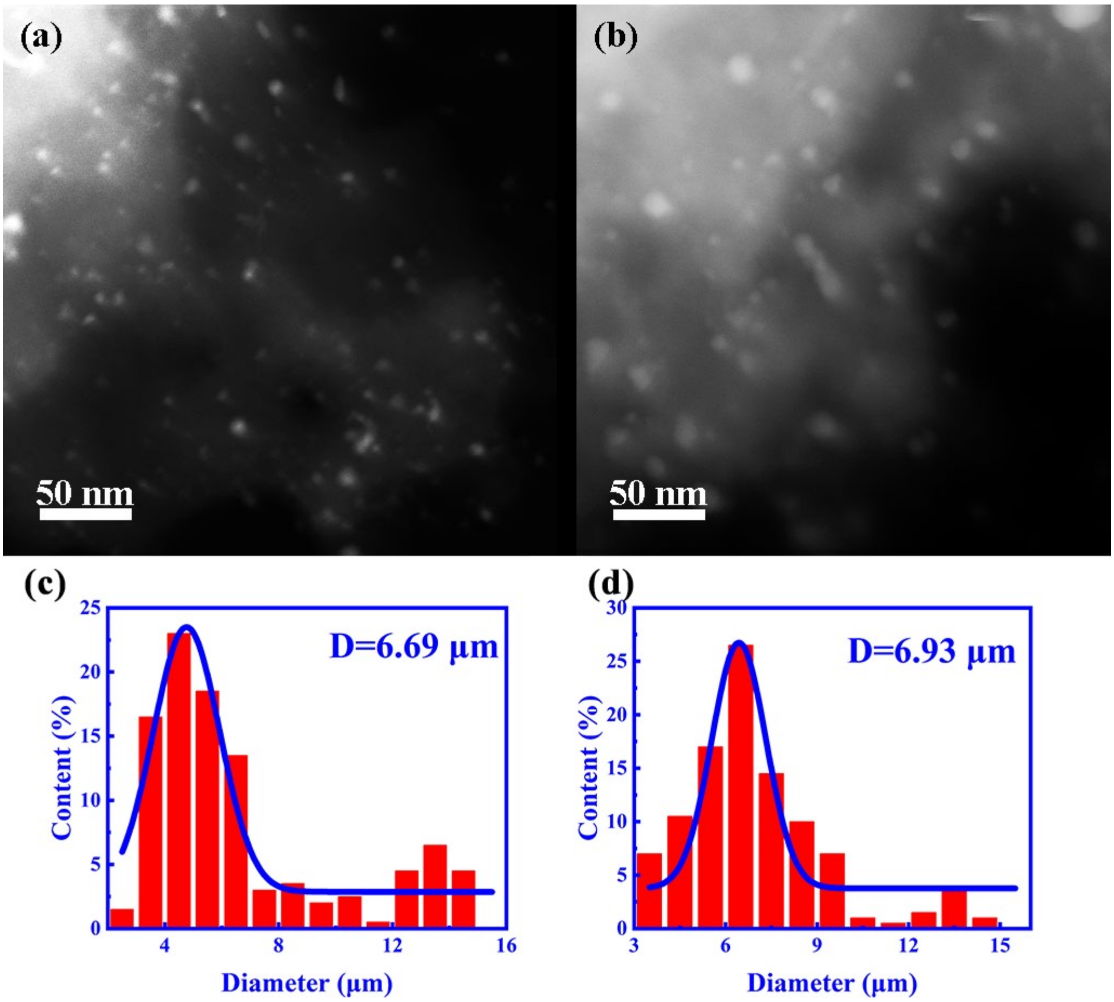

Figure S9. HAADF-STEM images of hcp-PtBi/MgO/Mg monolithic catalyst treated by (**a**) direct calcination at 600 ºC and (**b**) powder calcination at 900 ºC for 2 h in Ar. (**c**) and (**d**) are their corresponding NPs size distribution diagrams.

Table S1. The EDS results of hcp-PtBi/MgO/Mg monolithic catalyst.

Element	Weight %	Atomic %
O	51.10	60.95
Mg	32.67	25.64
Si	8.89	6.04
F	7.34	7.37
Totals	100.00	100.00

Table S2. The apparent activation energy (E_a) for 4-NP catalytic removal using other catalysts containing noble metals reported in other works.

**Catalyst**	**Support**	**Activation energy E_a (kJ/mol)**
PtBi	MgO	17.99
Pt_1Co_5/SAPO-34	SAPO-34	57.15
PtRu/C	C	51.5
$Pt_{0.67}Ni_{0.33}$/C	C	33.0
PtNi@SiO_2	SiO_2	54.76
Pt/SAPO-34	SAPO-34	61.88
Ag/Bi°-$BiVO_4$	$BiVO_4$	36.6
Au-Fe_3O_4	Fe_3O_4	1656

Table S3. The recyclability of hcp-PtBi/MgO/Mg catalyst and other catalysts prepared by various methods in other works.

Nanoparticles	Support	Method	Cycle Numbers
PtBi	MgO	PEO	50
PtNi	reduced graphene oxide	one-pot co-reduction method	4
PtPd	N-doped hollow carbon nanosphere	modified Stöber method	4
RuPd	H_xMoO_{3-y} Hybrid	impregnation method	5
PdAg	polydopamine-functionalized kaolin	co-reduction	10
PtRh	activated carbon	incipient wetness impregnation and simultaneous reduction	15
RhCo	nitrogen-doped porous carbon	thermal decomposition/reduction	5
Pd	iron-rich fly ash@fly ash-derived SiO_2	impregnation method	5
Ag	graphene oxide	solid-state chemical reaction method	7
Pt	micellar nanocomposites	chemical reaction method	5
Au	Hollow mesoporous CeO_2	colloidal deposition	8
Bi	N-doped reduced graphene oxide	one-pot hydrazine-involved hydrothermal process	5
